# An Interactive Computer Game for Improving Selective Voluntary Motor Control in Children With Upper Motor Neuron Lesions: Development and Preliminary Feasibility Study

**DOI:** 10.2196/26028

**Published:** 2021-07-28

**Authors:** Annina Fahr, Andrina Kläy, Jeffrey W Keller, Hubertus J A van Hedel

**Affiliations:** 1 Swiss Children's Rehab University Children’s Hospital Zurich Affoltern a.A. Switzerland; 2 Children’s Research Center University Children’s Hospital Zurich Zurich Switzerland; 3 Institute for Biomechanics, Department of Health Sciences and Technology ETH Zurich Zurich Switzerland

**Keywords:** virtual reality, game therapy, rehabilitation, augmented feedback, motivation, mobile phone

## Abstract

**Background:**

Computer game–based interventions are emerging in pediatric neurorehabilitation, as they can provide two key elements for motor learning—motivating environments that enable long-term compliance, which is particularly relevant for children, and augmented feedback for improving movement performance.

**Objective:**

The overall aim of this study is to develop an interactive computer play for children with upper motor neuron lesions to train selective voluntary motor control and give particular attention to motivation and feedback. We also aim to determine features that make games engaging, investigate which sensory feedback modality is noticed the fastest during play, develop an interactive game, and evaluate its feasibility.

**Methods:**

We identified engaging game features by interviewing 19 children and adolescents undergoing rehabilitation. By using a test version of the game, we determined the response times of 10 patients who had to react to visual, auditory, or combined feedback signals. On the basis of the results of these two subprojects, we developed and designed a game environment. Feasibility was studied in terms of the practicability and acceptability of the intervention among 5 children with upper motor neuron lesions.

**Results:**

The game features deemed the most important by pediatric patients were strategic gameplay (13/29, 45% of answers) and choice (6/29, 21%). While playing the game, an acoustic alarm signal (reaction time: median 2.8 seconds) was detected significantly faster (*P*=.01) than conditions with other feedback modalities (avatar velocity reduction: median 7.8 seconds; color desaturation: median 5.7 seconds). Most children enjoyed playing the game, despite some technical issues.

**Conclusions:**

The careful identification of game features that increase motivation and feedback modalities that inform children quickly led to the development of an interactive computer play for training selective voluntary motor control in children and adolescents with upper motor neuron lesions.

## Introduction

### Background

Patients with upper motor neuron lesions (ie, due to traumatic brain injury, stroke, or spastic cerebral palsy [CP]) exhibit a variety of motor impairments. These symptoms are typically categorized as positive or negative motor signs [1,2]. Positive motor signs are related to excessive muscle activity or movement, such as increased muscle tone and spasticity. Negative motor signs describe insufficient (control of) muscle activity or movement, such as weakness or loss of selective motor control. Reduced selective voluntary motor control (SVMC) is defined as “the impaired ability to isolate the activation of muscles in a selected pattern in response to demands of a voluntary posture or movement” [2]. Loss of SVMC can clinically manifest as impaired movement control and a multitude of involuntary movements (ie, unintended movements that co-occur with the performance of a voluntary task [3]), such as mass flexion or extension patterns, synergies of muscle activation (ie, obligatory grouped multi-joint movements), or mirror movements (ie, simultaneous, identical movements on the contralateral side) [4,5].

Although impairments in SVMC can be categorized according to the International Classification of Functioning, Disability, and Health into the domain of body function, they can interfere with movements of daily life activities and limit the patients’ independence. For example, children with unilateral spastic CP who exhibit mirror movements require more time for bimanual activities of daily life than peers without mirror movements, independent of their unimanual capacities [5]. In children with CP and lower limb impairments, gross motor function abilities are more strongly related to SVMC than other common impairments, such as muscle weakness or spasticity [6,7]. This relationship between SVMC and gross motor function was not only observed in cross-sectional evaluations but also in longitudinal studies, where an impairment in SVMC was found to be the strongest predictor of a less favorable course of gross motor function [8,9]. When focusing on ambulation, impairments in SVMC were associated with a reduction in gait speed due to decreased knee extension at initial contact, resulting in a reduced step length [10].

### Training Methods of SVMC

Multi-disciplinary rehabilitation programs aim to reduce children’s limitations in daily life activities to improve their participation. Despite the known importance of SVMC, only a few interventions have specifically aimed at improving selective control [11]. Robot-assisted ankle movement training implemented in a computer game was used to train the graded ankle dorsiflexion and plantarflexion [12]. After 18 training sessions over 6 weeks, children with spastic CP significantly improved their ankle SVMC. In another pilot trial, 4 children with spastic CP played a commercial video game, which was controlled by surface electromyography (sEMG) signals. It aimed to reinforce activity in a desired muscle and to reduce cocontraction of an agonist-antagonist muscle pair, thus training selective muscle activation [13]. In total, 3 of the 4 children could reduce muscle cocontraction during the game. A therapy program by Adler et al [14] specifically addressed mirror movements interfering with bimanual activities in children with unilateral spastic CP. However, exercises to suppress mirror movements emphasizing the independent use of both hands led to improved bimanual function without reducing the occurrence of mirror movements.

Although these interventions targeted certain aspects of SVMC (eg, improving joint movement control or reducing mirror movements), no approach covered all features of reduced selective motor control. We became interested in designing an intervention that would include training both aspects, improving accurate motor control while simultaneously reducing any sort of involuntary movement. On the basis of our experience with the application of rehabilitation technologies to improve upper and lower extremity functions in children and adolescents with neuromotor disorders [15,16], we started considering the design of a technology-assisted system combined with a game played in a virtual environment. Many technology-assisted interventions based on interactive computer play (ICP), exergames, and virtual reality (VR) have emerged to complement conventional therapies during rehabilitation [15,17,18]. The distinction between these terms describing similar technologies is not always easy, and VR is often used improperly [18,19]. ICP is defined as “any kind of computer game or VR technique where the individual can interact and play with virtual objects in a computer-generated environment” [20]. What distinguishes VR from more general ICP technologies is the immersive component of VR systems [21]. The purpose of exergames is to provide physical activity or exercise through interactive play [22]. An advantage of ICP is that it can provide key aspects relevant for motor learning during rehabilitation; for example, a playful environment increases motivation and thereby enables large numbers of repetitions, whereas the performance of movements can be improved based on augmented feedback [23].

### Objectives

Thus, the project’s overall aim was to develop an intervention for children with upper motor neuron lesions to improve SVMC by exploiting the advantages of ICP technology. Our basic idea was to develop an ICP that should train accurate joint movement control while providing immediate feedback when involuntary movements would occur.

Previous studies have already identified the properties of video games that are relevant when designing games because they affect motivation and player engagement, as follows: reward, optimal challenge, feedback, choice or interactivity, clear instructions, and socialization [24]. In addition to the theoretical background of game design principles and motivation, we wanted to consider the game design preferences of the target users. Augmented feedback should make the player aware of the occurrence of involuntary movements while playing an attention-demanding ICP. Therefore, special attention should be given to the question of how such *warning* feedback is ideally provided. Finally, we aimed to follow up the ICP design process with a small feasibility trial. Therefore, we formulated the following specific research questions for the development of an ICP to train SVMC: (1) which features of commercial games do children and adolescents undergoing neuro-orthopedic rehabilitation like, (2) which modality of feedback indicating a negative occurrence is perceived most easily by these patients while playing an ICP, and (3) is it feasible to use the new ICP as an intervention for children and adolescents with impaired SVMC due to an upper motor neuron lesion?

## Methods

### Study Overview

To develop an ICP that should motivate children and adolescents with impairments in SVMC to practice for a longer time, we conducted three subprojects: (1) identifying motivating game features by conducting interviews; (2) identifying which *warning* feedback signal is detected the quickest while playing the game; and (3) based on the results from these subprojects, the ICP is developed and its preliminary feasibility is evaluated.

This game development study did not fall under the Human Research Act, which was confirmed by the Ethics Committee of the Canton Zurich through two clarifications of responsibility (Req-2019-01005 and Req-2019-00623).

### Motivating Game Features

For the survey, we recruited children and adolescents regularly playing games on smartphones or tablets undergoing neuro-orthopedic inpatient rehabilitation at the Swiss Children’s Rehab. The participants and their parents were verbally informed about the interview. Verbal consent was obtained from both before the structured interviews were conducted. In the interview, we gathered ideas and explored the participants’ opinions with the following open-ended questions (in German):

- What are your favorite games?
- What do you like about them? Several answers were possible for each game.
- What feature is the most important for you and would need to be included if you could design your own game?

We asked participants to think of games for smartphones or tablets rather than for game consoles. The latter have extensive control options (eg, many keys or joysticks) that allow complex user interactions. Smartphone or tablet games usually include simpler control modes, which are in line with our idea of training SVMC using basic isolated joint movements to control the ICP. If participating children or adolescents had difficulties in naming game features spontaneously, we provided suggestions including graphics, actions, shooting, the story, music, being challenged, puzzles, different strategies, character development, large game world, and multiplayer.

### Feedback Modalities

#### Participants

For the second subproject, we again recruited children and adolescents undergoing neuro-orthopedic inpatient rehabilitation. They had to be able to use the arrow keys and press the spacebar on a regular computer keyboard. The exclusion criteria were severe visual or auditory impairments. All participants and their parents were informed orally and written about the protocol. Parents and adolescents aged ≥14 years provided written informed consent. All participants provided verbal informed consent.

#### Test Game

**Figure 1:**
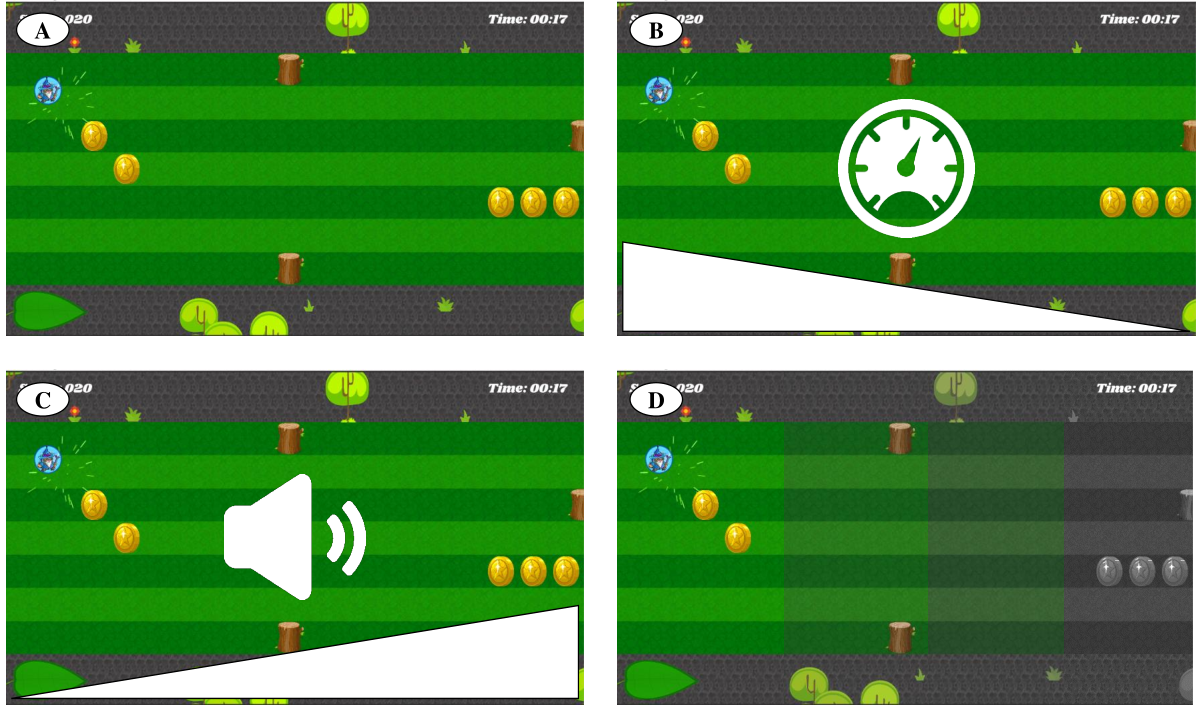
Preliminary game environment and schematic illustration of feedback modes for testing different feedback signal modalities. (A) Game environment in which the avatar had to be navigated up and down. (B) Baseline mode where the avatar’s speed was reduced. (C) In the acoustic mode, the feedback signal was an alarm sound of increasing sound volume. (D) Illustration of the conversion from color to gray scale in the background color mode.

We created a reaction time (RT) task to investigate in which modality a *warning* feedback signal is noticed most easily while playing the game. Thus, in this experiment, the augmented feedback signal did not inform the player about undesired comovements, but the signal was simulated and participants had to detect it while playing. The game environment was a preliminary version of the ICP programmed in Unity (Figure 1). The goal of the game was to move an avatar up and down on the screen to collect coins and avoid obstacles. The avatar was controlled with arrow keys on a commercial computer keyboard to provide similar conditions for all participants.

**Figure 2:**
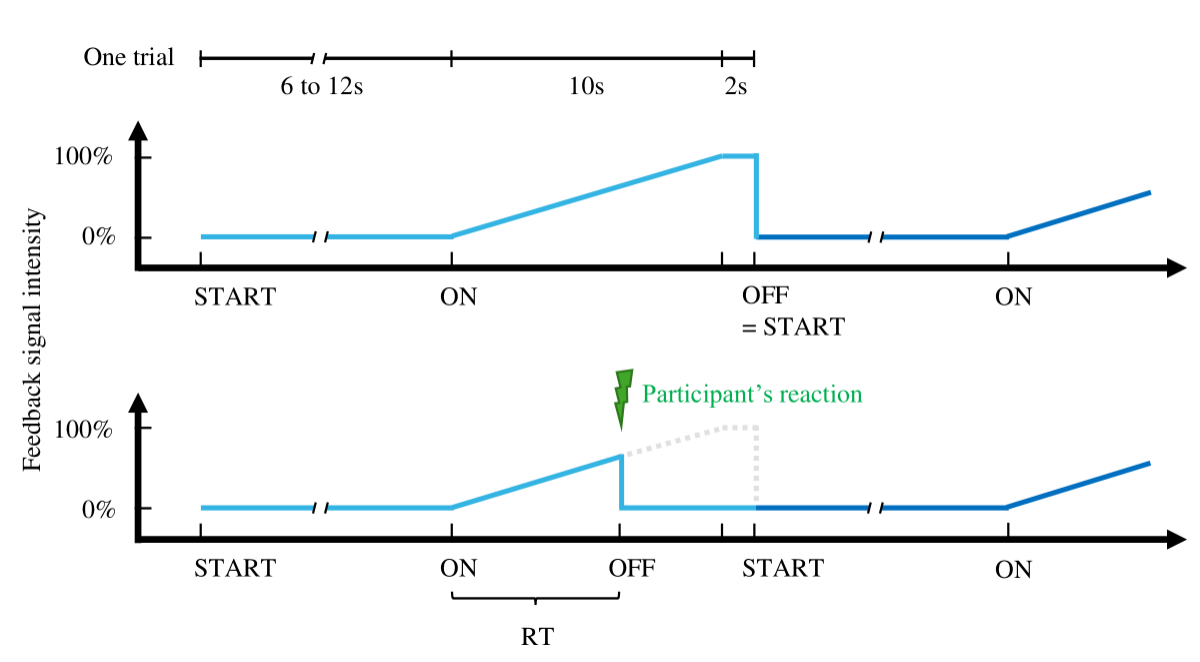
The upper part shows the organization of one trial, illustrating the start of the trial, the feedback initiation (ON) and increment, and the start of the next trial. The lower part of the figure illustrates the changes that occurred as soon as the participant responded to the feedback signal and how the reaction time was defined. RT: reaction time.

We investigated three modes of gradable feedback signals. Feedback 1, *Baseline*: no additional signal apart from reducing the avatar’s speed, which had the aim of facilitating the game to enable focusing on the purpose of the game again; Feedback 2, *Acoustic*: playing an alarm sound; and Feedback 3, *Background color*: converting the background from color to grayscale (Figure 1). We tested four conditions: (1) Feedback 1 alone, (2) Feedback 1 and 2, (3) Feedback 1 and 3, and (4) Feedback 1, 2, and 3. The feedback signal was introduced randomly between 6 and 12 seconds after a trial had started and gradually increased from 0% to 100% feedback signal intensity (ie, speed reduction from normal game speed to completely static, increase in sound volume of the alarm, and color desaturation to complete gray scale) in 10 seconds. It remained for 2 seconds at 100% signal intensity before the test automatically continued with the next trial. The participants’ task was to press the space bar to turn off the signal as soon as they had detected the feedback signal while continuing to play the game. In this case, the time remaining until the next trial started was normal game play, such that feedback detection success had no influence on the duration of the game. A schematic illustration of the feedback simulation trial design is provided in Figure 2.

#### Procedures

The participants were instructed to collect coins by moving the avatar with the arrow keys and pressing the space bar when they noticed one of the feedback signals. During the familiarization phase (approximately 2 minutes), they could try the game and experience each feedback condition once. Each condition was tested five times, resulting in 20 trials. Trials were completed in a blocked randomized order [25] to control for fatigue. The trials were tested consecutively such that the test appeared to be a continuous game, which lasted approximately 7 minutes.

#### Data Analysis and Statistics

The main outcome was the RT, extracted from the game that logged the onset of the feedback signal and when the participant pressed the space bar. RTs below 0.1 seconds were excluded because they likely reflected pressing by chance (healthy adults did not achieve faster RTs). The average RT per person was calculated for each condition. Not reacting to a feedback signal was counted as a missed response and led to an entry of 12 seconds as the RT (as penalty).

We used the R statistical package (version 3.4.4; R Foundation for Statistical Computing). Normality of the data was evaluated using Shapiro-Wilk tests and by visual inspection of the Q-Q plots. As not all data were normally distributed, conditions were compared using a nonparametric Friedman test with pairwise Wilcoxon signed-rank tests. The significance level α was set at .05, with a Bonferroni correction for multiple comparisons.

### Game Design and Feasibility Trial

#### SVMC Runner Game

**Figure 3:**
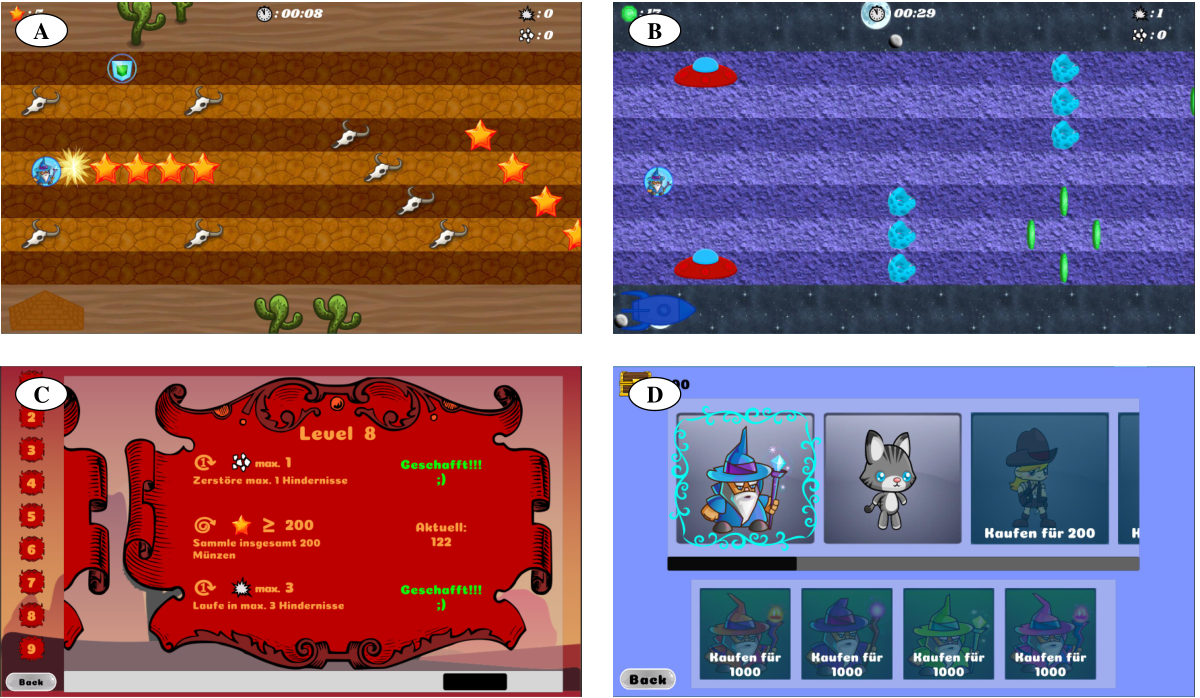
Game environment. The purpose of the game was to collect stars or coins and avoid the obstacles by moving the avatar up and down on the screen. (A) This screenshot from a game level shows a shield power-up in the top-left corner. After collecting it, the avatar was protected from obstacles for the next 10 seconds. (B) This level had a different theme, although the game elements stayed the same. (C) An example of challenges in one level. Each challenge required a different focus. (D) Players could use the collected coins to personalize the avatar.

The game environment was programmed in Unity (version 2019.3.0f6, Unity Technologies) and included sounds and background music. The game design was based on the results of the two previous subprojects, hence the survey on favored game features and the investigation of feedback modalities. A video of the game can be seen in Multimedia Appendix 1. The goal of the game was to move an avatar up and down on the screen to collect coins and avoid certain obstacles (Figure 3). The avatar’s horizontal position was fixed while the game environment moved at a steady pace of shifting by one screen width in approximately 6.5 seconds (ie, an object appearing on the right side of the screen required 6.5 seconds until it disappeared on the opposite side). As soon as involuntary movements occurred, an auditory augmented feedback signal made the player aware of the occurrence of involuntary movements (based on results from the second subproject). The signal volume was graded according to the extent of the involuntary movements. Furthermore, the avatar’s speed gradually slowed, relative to the involuntary movements. This response to involuntarily occurring movements was implemented to make game play temporarily easier. It should enable the player to focus again on the game and reduce the occurrence of comovements, because these often appear in conditions of increased effort [3].

The game included six thematically different game environments (*worlds*). The six worlds covered 54 levels of increasing difficulty (eg, from shorter to longer duration; from a few, small, static obstacles to more, larger and moving obstacles; and from no enemy to multiple enemies). Each player had their own profile. Initially, only the first level was playable, whereas the others remained locked. After successfully finishing a level, the next higher level became unlocked. At the onset of each level, certain challenges (eg, collecting a certain amount of coins) that the player needed to achieve were presented. Players were provided three challenges per level that required them to adopt (varying) strategies (Figure 3). Advancing to a new world needed fulfillment of at least 80% of these challenges. The players experienced choice as they could freely decide which challenge or challenges they wanted to tackle. Variability was further enhanced by adding power-ups (ie, objects that add temporary extra abilities to the game character, such as increasing speed, a shield protecting against enemies; Figure 3) to the environment. Once enough coins had been collected, they could be used to personalize the avatar (Figure 3).

In addition, we considered the visual presentation of the game to be important. The appealing and motivating effect of visual features had to be balanced against the risk of confusion or distraction from the basic task, which can be particularly important for children with a recently acquired brain injury. With glasses simulating a strong visual impairment (provided by the *Swiss National Association of and for the Blind*), we verified that contrasts between essential gaming elements were high, as more than 50% of the children with CP experience visual impairments [26].

#### Game Controllers

**Figure 4:**
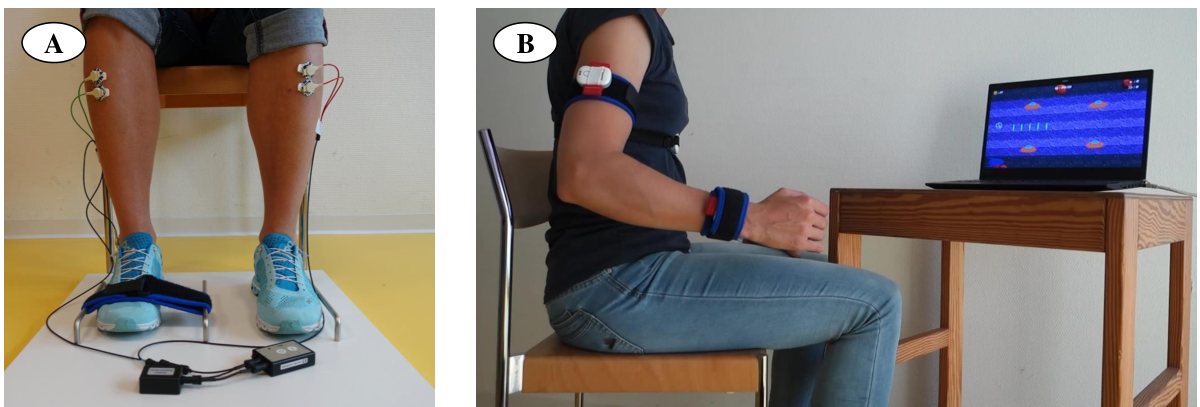
Game controllers. (A) Electromyography-based control of the game via activation of the right tibialis anterior muscle (green cables). Mirror activity on the left side (red cables) would trigger the feedback signal. (B) ArmeoSenso with its three sensors (chest, upper arm, and lower arm). In this example, the game was controlled by elbow flexion and extension without simultaneously moving the shoulder (ie, shoulder abduction would lead to a warning feedback signal).

We pursued the following two control strategies for the ICP to improve SVMC: (1) a strategy based on muscle activity and (2) a strategy based on joint movements (Figure 4). The first approach trained the selective activation of a target muscle (group) while reducing activity in another muscle (group) that should remain inactive (similar to Rios et al [13] and Yoo et al [27] but not restricted to agonist-antagonist muscle pairs). The sEMG signals of these two selected muscle groups were recorded using a varioport-e device (Becker Meditec) at a sampling frequency of 1000 Hz. The sEMG signals were transferred via Bluetooth to the ICP computer, where they were filtered (exponential smoothing with a smoothing factor of 0.0015, followed by taking a moving average with a window size of 10 frames) and fed into the game. The avatar was steered up by increasing the activation of the target muscle (group), while lowering the sEMG activity caused a downward movement of the avatar. The *warning* feedback signal was triggered by activity in the muscle (group) that should remain inactive.

For the second approach, we used the ArmeoSenso system (version 1.0; Hocoma AG). This motion capture system was developed for arm rehabilitation and included three wearable inertial measurement units to detect movements of one arm and trunk movements. Although it was originally intended for unilateral upper extremity rehabilitation, it could also be used to track lower extremity movements (unilateral). Joint angles delivered by the inbuilt software were transferred via the user datagram protocol to the ICP computer and served as input signal for the game to train selective movement of the chosen target joint and record involuntary movements in another joint. The upward and downward movements of the avatar were caused by moving the target joint in an upward and downward direction, respectively. Movements of the joint that should not move activated the *warning* feedback signal.

#### Pilot Tests

Before testing the game in children and adolescents with impairments in SVMC, we performed pilot sessions in healthy adults and children to identify and resolve bugs in the game design and explore different hardware configurations.

#### Participants

For the feasibility trial, we recruited children and adolescents with impaired SVMC due to upper motor neuron lesions. Improving SVMC was identified by their physical or occupational therapist as a reasonable rehabilitation goal of their inpatient stay at the Swiss Children’s Rehab. All participants and their parents were informed verbally and in writing about the protocol and provided written informed consent.

#### Study Design

The study encompassed six sessions of 40 minutes scheduled over 2 weeks. In the first two sessions, we tested each control system (one system per session in randomized order). In the following four sessions, we used the more appropriate system. Together with the participants’ physical or occupational therapist, who treated each participant, we determined the muscle group or movement that had to be trained (target joint) and the involuntary muscle activation or movement that needed to be suppressed.

#### Outcomes

Study participants were characterized by age, sex, diagnosis, and SVMC impairment measured using the Selective Control Assessment of the Lower Extremity or Selective Control of the Upper Extremity Scale [28-30]. We evaluated the domains’ practicability and acceptability, as described in the model of clinical utility by Smart [31]. We recorded any technical issues related to setting up the ICP, playing the game, and whether the game’s purpose was clear to the participant. To compare the hardware approaches and hints on which strategy to pursue further, participants decided on their preferred system and the therapist indicated which system was more appropriate in terms of reaching the therapy goal. In view of using the ICP in an intervention study in the future, we assessed the time the participant was actively playing. Therefore, the game logged the time when levels were played, excluding all breaks.

After each session, participants were verbally asked whether they enjoyed the game (three-point Likert-scale with answers “no,” “a little,” and “a lot of fun”), whether they would like to play again, and if they experienced any discomfort or harm.

#### Procedures

Each session started by attaching the sensors or electrodes and calibrating the game, which was accomplished by moving the joint once through the active range of motion or maximally contracting the muscle. In the sEMG mode, the limb was then strapped to a custom-made board with loops for Velcro straps to prevent any movement of the target joint. We decided to control the game by isometric muscle activation after pilot tests, which revealed that when movement was allowed, the joint was mostly held in a position at the end of the range of motion. This led to pain only after a short period of playing. We used standardized, tutorial-like, step-by-step instructions to guide participants in the game environment. Participants decided on their own level of play and which challenge to tackle. In the first two sessions, played with the different control systems, participants started at level 1 with separate game profiles. This enabled participants to start at the easiest level with both control systems. Sessions 3-6 continued progressively with the system the therapist deemed more appropriate (also considering the participant’s opinion).

## Results

### Motivating Game Features

We interviewed 19 inpatients from our rehabilitation center (9 males; mean age 14.3, SD 2.5 years; range 8.0-17.0 years). The most common diagnosis was traumatic brain injury (n=6). Participants mentioned 29 games, of which Minecraft was mentioned three times, followed by 4 Elements, Clash of Clans, and Fortnite, each mentioned twice. They considered requiring a strategy to play the game as the most important feature of the game (13/29, 45% of all answers), followed by having multiple *options* (6/29, 21%). The term *options* encompasses that the game allows the player to decide between multiple possibilities to achieve the goal. Further characteristics that participants liked were the possibility of playing in a multiplayer mode, power-ups, and character evolution (Table 1). A full list of answers can be found in Multimedia Appendix 2.

**Table 1:** Features of the participants’ favorite games. Several game features could be mentioned per game (n=29).

Game feature	Answers, n (%)
Multiplayer	13 (45)
Strategic gameplay	13 (45)
Power-ups	11 (38)
Multiple options	8 (28)
Character evolution	7 (24)
Creativity	3 (10)
Fighting	3 (10)
Animations	2 (7)
Shooting	2 (7)
Speed	2 (7)
Timing challenge	2 (7)
Machines	1 (3)
Simple idea	1 (3)
Sport	1 (3)

### Feedback Modalities

**Figure 5:**
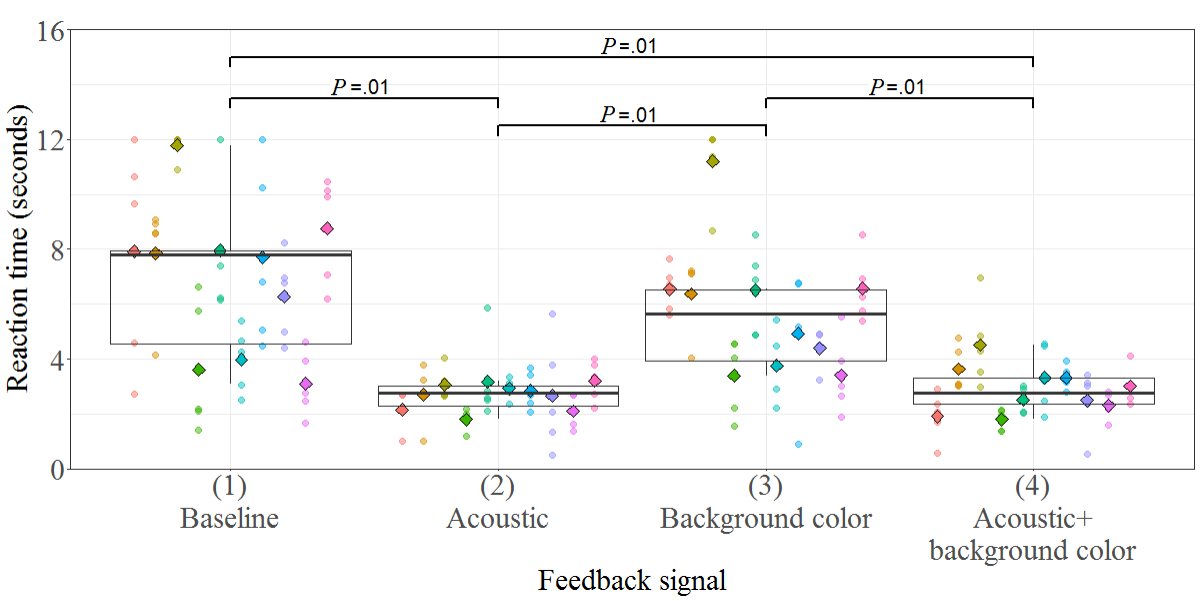
Boxplots of the reaction times for each feedback mode (1-4). In the baseline condition, only the speed of the avatar was reduced. In the other conditions, velocity reduction was combined with an additional feedback signal, that is, an acoustic alarm or a change of the background color. The colors represent individual participants. The small circles show the reaction time of each individual trial, whereas diamonds show the mean reaction time over all 5 trials per participant.

A total of 10 children and adolescents (male: n=9; age: mean 12.6 years, SD 3.5 years; range 7.3-16.3 years) participated. The participants’ diagnoses were bilateral spastic CP (n=5; Gross Motor Function Classification System I-IV and Manual Ability Classification System I-II), sensory-motor neuropathy (n=1), epilepsy (n=1), congenital hemiplegia (n=1), anti–N-Methyl-D-aspartate receptor encephalitis (n=1), and intracranial hemorrhage (n=1). The RTs are shown in Figure 5. They differed significantly between the conditions (*χ*^2^_3_=26.2; *P*<.001). Pairwise comparisons revealed that the RT was significantly longer (*P=*.01) in conditions without the acoustic feedback signal (conditions 1 and 3) than in conditions that included the alarm sound (conditions 2 and 4). RT tended to be longer (*P*=.06) in the baseline condition (1), where only the velocity was reduced, compared with the background color condition (3). RTs in conditions 2 and 4 did not differ (*P=*.99).

Although none of the participants missed responding in any acoustic feedback signal trial, 14% (7/50) of responses in total were missed in the baseline condition of only velocity reduction, whereas 6% (3/50) were missed in the background color condition.

### Feasibility Trial

The ICP was piloted in 11 sessions with healthy participants; 5 children and adolescents with impaired SVMC were recruited, and their characteristics are shown in Table 2. One participant (ID 2) stopped the study after two sessions owing to the premature end of rehabilitation because of the COVID-19 pandemic.

**Table 2:** Characteristics of participants in the feasibility study (n=5).

ID number	Age (years)	Sex	Diagnosis	SVMC^a^ total (points)	SVMC target joint (points)	System	Target joint or muscle group^b^	Involuntary movement
1	7.8	Female	Bilateral spastic CP^c^, GMFCS^d^ III	5^e^	0^e^	sEMG^f^	Ankle dorsiflexion	Ipsilateral knee extension
2	13.0	Male	Bilateral spastic CP, GMFCS II	8^e^	0^e^	ArmeoSenso	Knee flexion and extension	Trunk flexion and extension
3	12.7	Female	Bilateral spastic CP, GMFCS III	7^e^	1^e^	sEMG	Knee extension	Contralateral knee extension
4	12.5	Female	Traumatic brain injury	16^e^	1^e^	sEMG	Ankle dorsiflexion	Contralateral ankle dorsiflexion
5	16.1	Male	Unilateral spastic CP, MACS^g^ III	20^h^	2^h^	ArmeoSenso	Shoulder abduction and adduction	Ipsilateral elbow flexion and extension

^a^SVMC: selective voluntary motor control; SVMC total: total Selective Control Assessment of the Lower Extremity (SCALE) score (maximum of 2 points per joint; total of 20 points) or Selective Control of the Upper Extremity Scale (SCUES; maximum of 3 points per joint; total of 30 points); SVMC target score: SCALE or SCUES item scores for the target joint that was practiced with the game.

^b^Refers to the target joint when the ArmeoSenso system was used and to the target muscle group when the electromyography-based system was used.

^c^CP: cerebral palsy.

^d^GMFCS: gross motor function classification system (it standardizes the classification of gross motor function, emphasizing the trunk control and walking ability of children with cerebral palsy [32]. Level 1 means that children perform all the activities of neurologically intact children of the same age, allowing for slight limitations in speed and quality of movements. Level 5 means that children exhibit difficulties in head and trunk control in most positions or in achieving any voluntary control of movement at all).

^e^Selective voluntary motor control assessed with the Selective Control Assessment of the Lower Extremity.

^f^sEMG: surface electromyography.

^g^MACS: manual ability classification system (it classifies how children with cerebral palsy handle objects in daily activities [33]. Level 1 means that the child can handle objects easily and successfully, whereas children at level 5 do not handle objects at all).

^h^Selective voluntary motor control assessed with the Selective Control of the Upper Extremity Scale.

In one case, the ArmeoSenso was favored (ID 5) and also once, the participant and therapist clearly preferred the electromyography-based system (ID 1), because steering the game with the ArmeoSenso proved to be challenging in the case of a small active range of motion. No comparison of systems was possible for the other participants. In two cases, because the unilateral ArmeoSenso system is not able to track mirror movements (IDs 3 and 4), we could only use the sEMG system; in the third case, the patient withdrew (ID 2), but the motion capture system was working reliably until then.

It took more time than expected to set up the ICP until game control functioned reliably, because we encountered difficulties in conducting and standardizing the calibration. Recalibration was necessary more frequently than expected, because problems occurred with steering. Participants were actively playing for 18.3 (SD 5.4) minutes per session on average (range 9.8-30.4 minutes) with training time increasing over the course of the study (15.8 minutes in first session vs 20.3 minutes in fifth session).

**Figure 6:**
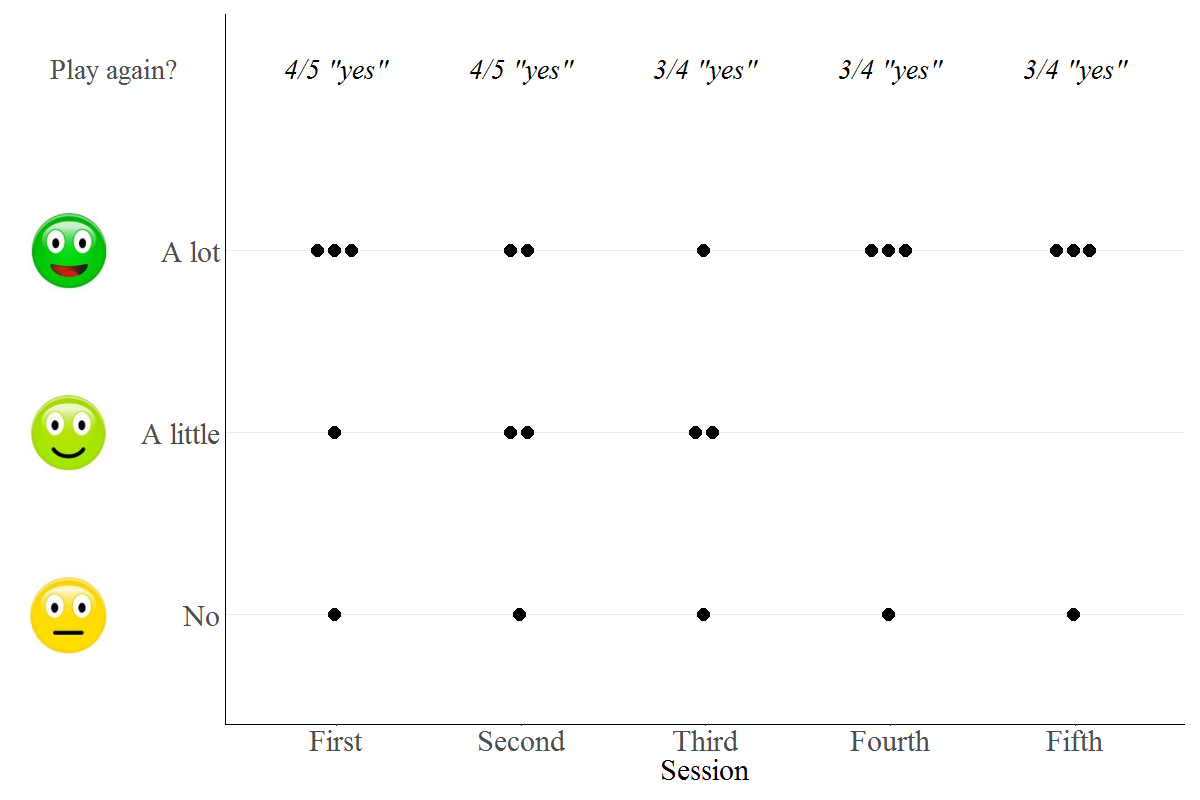
Participants’ ratings of enjoyment. Scores indicated how much they liked the game and whether they would like to play it again. Only sessions with the more appropriate system are shown.

Only one participant answered having experienced “no fun at all” (ID 5), while having “a lot of fun” represented the majority of answers. ID 5 remained uninterested over all sessions, whereas two that were a little skeptical in the beginning became more enthusiastic over time (Figure 6). None of the participants reported any discomfort or harm.

## Discussion

### Principal Findings

This study describes the development of an ICP to specifically enhance SVMC in children and adolescents. The game-based intervention trains movement control while simultaneously providing feedback to reduce involuntary movements. We designed the game environment based on the favorite game features that children reported in a survey. Subsequently, we tested various feedback conditions to identify the auditory signal as the modality that patients perceived the fastest. An ensuing investigation of usability revealed that children enjoyed the game despite some technical issues.

### Motivating Game Features

Interestingly, the features mentioned most frequently, strategic gameplay, and having multiple options depend on each other. If a game provides many options, the player needs a strategy to succeed, and vice versa, players can only develop a strategy if they are provided with more than one option. These features are comparable with the interdependent construct choice and interactivity, which are considered key principles of game design to increase motivation [24]. Strategic gameplay was also one of the motivators of video games for therapy mentioned by children with hemiparesis in a focus group [34]. Furthermore, variability, challenge, and competition were identified as common elements that increased motivation in a study investigating the effect of several ICP environments on motivational behaviors in children with CP [35]. Although variability was not a specific response in this study, the features of multiple options, power-ups, and character evolution introduce variability and help to avoid the fact that a game looks the same every time it is played.

Multiple options were incorporated into the game design in several ways. The game did not follow one particular trajectory with the game avatar but allowed different ways through the environment. Furthermore, players could choose the level to play and which challenge to tackle on their own. Presenting three challenges for each level forced the player to develop and use varying strategies to fulfill each of them. Finally, we included power-ups, the possibility to personalize the game character, and varied the visual appearance of the game with a thematic design of the worlds.

### Feedback Modalities

Unlike common simple RT tasks, this study investigated a dual-task situation. While participants focused on collecting coins and avoiding obstacles, the feedback signals had to attract their attention. The game already provided many visual inputs, which might have complicated the detection of other visual stimuli (such as reducing speed or changing background color) due to overloading visual perception [36]. In particular, during parts of the game with many obstacles, the visual load could have been high. Although the game sounds and background music were not relevant for playing the game, the alarm sounds were easy to distinguish. The RTs and their variability were considerably lower in conditions with an auditory feedback signal than in conditions with only visual signals. This suggests that the alarm sound had the highest contrast to the game environment and attracted attention most easily.

### Feasibility Trial

On the basis of most participants expressing moderate-to-high enjoyment and no discomfort, the ICP intervention can be considered acceptable. One child’s spontaneous comment (“I cannot reach this [next] world with only one more session, can I come more often?”) revealed that the design of the game environment created long-term motivation and engagement. These results confirm that game-like interventions can lead to high levels of compliance and motivation [37]. Increased fun and interest in the game-based intervention was also shown in a study directly comparing conventional ankle dorsiflexion training with an ICP protocol [38]. High levels of motivation increase the time dedicated to an intervention and would thereby contribute to the success of a therapy, which depends on adherence over an extended period. We can only speculate why one participant did not like the ICP based on our observations during the training sessions. The target movement proved rather difficult for this participant, and the ICP did not allow control of the game by the compensatory movement pattern he usually used. This frustrated him, and it seemed that he was not motivated to practice something challenging in general. Lower levels of motivation to persevere in challenging situations have already been reported in children with CP [39]. We have to be aware of such personal reactions and need to seek solutions to prevent frustration, resulting in loss of compliance.

Some issues encountered with the game calibration interfere with the practicability of the ICP. The time needed to solve these issues automatically reduced the active training time because the overall duration of sessions was determined by the clinic schedule. Moreover, the occurrence of technical problems sometimes seemed to coincide with lower levels of enjoyment. Difficulties arose during the first sessions and resolved during later sessions. This also finds reflection in a longer active training period during later sessions. Further measures are needed to increase the active training time because we think that 15-20 minutes is a rather low amount of training during sessions lasting 40 minutes. Adult patients with stroke engaged in physical activity for 60% of the time during a physical therapy session [40]. Activity rates during technology-assisted rehabilitation were higher than those during conventional therapy [41]. However, we must acknowledge that exploiting the game features we included to increase motivation and engagement (ie, choice, challenges, and personalization) took time. Moreover, by selecting a single target joint, our intervention was very specific, and the active time was fully dedicated to train one particular movement. At the same time, this might cause children to need more breaks because switching between different tasks for a change was not possible.

The comparison of hardware systems (game controllers) did not reveal a clear favorite but showed that both systems were required. The use of the sEMG system was obligatory to address mirror movements during training because ArmeoSenso cannot track contralateral movements. The sEMG system was also superior in the case of a small active range of motion, where motion tracking with ArmeoSenso failed. However, ArmeoSenso was particularly useful for recording trunk movements, where the selection of one representative muscle would be difficult. These findings further strengthen our view of an individualized approach to improving SVMC in children and adolescents with upper motor neuron lesions.

### Limitations

With the survey in the first part of our study, we only assessed the subjective preferences of children and adolescents about game features, assuming that the preferred game elements are motivating. However, it would require a longitudinal study in which these features were systematically manipulated to investigate their influence on long-term engagement.

A limitation of the investigation of feedback modalities is that the game was played with the arrow keys and not with the intended way of steering through muscle activation or joint movement. Nevertheless, we would not expect the results to be different because both steering modes require coordination of one’s movement according to what is displayed in the game. Using the arrow keys might have shortened RTs (for all feedback modalities), as this mode of control is likely more common and therefore less challenging than controlling a game by body movements.

Although the overall aim was to develop an intervention for patients with reduced SVMC due to an upper motor neuron lesion, we recruited children with various diagnoses undergoing neuro-orthopedic rehabilitation for the first two subprojects, as we considered these research questions unrelated to SVMC. An advantage of investigating a more general population of children undergoing rehabilitation is the higher generalizability of the results to the ICP design for other pediatric patient groups.

We could not evaluate the preliminary effectiveness of the ICP to determine its appropriateness, although we had intended to do so with an assessment integrated with the ICP, similar to a previously developed assessgame [42,43]. Difficulties in standardizing the calibration prevented comparability between sessions or after recalibration. We are planning a single-subject research design study to evaluate the effectiveness of the game in improving motor control and reducing the occurrence of involuntary movements compared with regular multimodal rehabilitation in children and adolescents with impairments in SVMC. After showing effectiveness in a clinical setting, a future plan could be to implement the intervention in a home-based setting. It would also be interesting to investigate whether potential improvements in SVMC are meaningful to children and translate to improvements in activities of daily life.

### Conclusions

This study revealed that strategic gameplay and having multiple options are favorable and likely motivating features of games for children and adolescents undergoing rehabilitation. This indicates that they prefer to choose their own strategies among several options to influence the course of a game. We incorporated these findings in the design of an ICP to train SVMC, as well as the finding that an acoustic warning signal was perceived more quickly than the other modalities that we tested. Feasibility testing revealed that participants enjoyed playing, but some technical issues impeded user experience and should be addressed to optimize practicability. The results of this study could be considered by designers of other interactive computer games if long-term engagement of children, such as during a rehabilitation period, is needed.

## Appendix

Multimedia Appendix 1 Video of the game. This shows the avatar being navigated through the game environment, collisions with obstacles, and the effects of a power-up.

Multimedia Appendix 2 List of all answers during the interview.

## Abbreviations

CP: cerebral palsy
ICP: interactive computer play
RT: reaction time
sEMG: surface electromyography
SVMC: selective voluntary motor control
VR: virtual reality
