# Supplementary material for: An Interactive Computer Game for Improving Selective Voluntary Motor Control in Children With Upper Motor Neuron Lesions: Development and Preliminary Feasibility Study
Source: JMIR Serious Games. 2021 Jul 28;9(3):e26028. doi: 10.2196/26028 (PMC8367178; doi:10.2196/26028)
Supplement: Multimedia Appendix 2 [file games_v9i3e26028_app2.pdf]

Multimedia Appendix 2
Full list of answers

What are your favorite games?

Name of the game	Answers, n
Minecraft	3
4 Elements	2
Clash of Clans	2
Fortnite	2
Brothers in Arms	1
Bus Simulator	1
Chess	1
Clash Royale	1
Critical Ops	1
Doodle Jump	1
Dune	1
Fifa	1
Football Strike	1
Fun Run	1
Geometry Dash	1
Hay Day	1
Helix Jump	1
Merge Plane	1
Paper.io 2	1
Plants vs. Zombies	1
Rush Hour	1
Sims	1
Sonic Dash	1
Subway Surfers	1

What feature is the most important for you and would need to be included if you could design your own game?

Game feature	Answers, n (%)
Strategic gameplay	13 (45)
Multiple options	6 (21)
Multiplayer	2 (7)
Shooting	2 (7)
Speed	2 (7)
Timing challenge	2 (7)
Machines	1 (3)
Sport	1(3)
